# Supplementary material for: Azobenzene-containing liquid crystalline composites for robust ultraviolet detectors based on conversion of illuminance-mechanical stress-electric signals
Source: Nat Commun. 2021 Aug 12;12:4875. doi: 10.1038/s41467-021-25178-2 (PMC8360969; doi:10.1038/s41467-021-25178-2)
Supplement: Supplementary file 1 — Supplementary Information [file 41467_2021_25178_MOESM1_ESM.pdf]

Supplementary information

Azobenzene-Containing Liquid Crystalline Composites for Robust Ultraviolet Detectors Based on Conversion of Illuminance-Mechanical Stress-Electric Signals

Xiaoxiong Zheng[1, 2], *Yining Jia*[1, 2] & *Aihua Chen*[1, 2]*

[1] School of Materials Science and Engineering, Beihang University, No. 37 Xueyuan Road, Haidian District, Beijing 100191, P. R. China
[2] Beijing Advanced Innovation Centre for Biomedical Engineering, Beihang University, No. 37 Xueyuan Road, Haidian District, Beijing 100191, P. R. China

Correspondence to Aihua Chen (E-mail: chenaihua@buaa.edu.cn)

Content

Section 1: Synthesis of block copolymers

Section 2: Supplementary figures and tables

Section 3: Calculation of ultraviolet protection factor (UPF)

References

Section 1: Synthesis of block copolymers

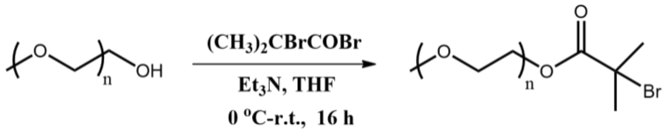

Scheme 1. Preparation of the PEO macroinitiators.

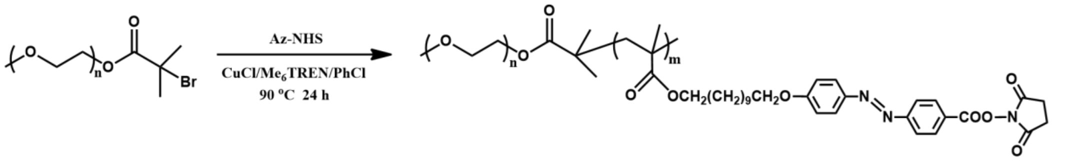

Scheme 2. Preparation of the block copolymers

Preparation of PEO macroinitiators. The synthesis of the macroinitiators is shown in Scheme 1, similar to the previous study.[1] A solution of 0.276 g (1.2 mmol) of α-bromoisobutyryl bromide in 5 mL of dry tetrahydrofuran (THF) was added to a mixture of 0.152 g (1.5 mmol) of triethylamine and 2 g (1.0 mmol) of PEO-OH with an M_n of 2000 in 20 mL of THF at 0 °C, and then the mixture was stirred for 16h at ambient temperature. After the mixture was filtered, half of the solvent was evaporated, and the PEO macroinitiator (PEO-Br) was precipitated into cold ether. After dissolution in ethanol, the solution was stored in a refrigerator to recrystallize the product. Yield: 1.75 g (80%). M_n(GPC)=2200, M_w/M_n =1.04.

Preparation of the block copolymers. The synthesis of the block copolymers is shown in Scheme 2. 13.4 mg (0.136 mmol) of Cu(I)Cl, 300 mg (0.136 mmol) of PEO macroinitiators, and 1940 mg (3.356 mmol) of the reactive azobenzene-containing monomer[2] were mixed in a 50 mL Schleck flask, degassed and filled with nitrogen. A 109.0 µL (94 mg, 0.409 mmol) of Me_6TREN in 4 mL of chlorobenzene was added through a syringe. The mixture was degassed three times using the freeze-pump-thaw procedure and sealed under vacuum. After 30 min stirring at room temperature, the Schleck flask was placed in the preheated 90°C oil bath for 24h. The solution was passed through an acidic Al_2O_3 column with THF as eluent to remove the catalyst. The yellow filtrate was concentrated under reduced pressure and reprecipitated into ether and ethanol. The yellow polymer was collected by filtration and dried under vacuum. Yield: 1860 mg (75%). M_n(NMR)=18156, M_n(GPC)=13572, M_w/M_n =1.13, as shown in Figure S1.

Section 2: Supplementary figures and tables

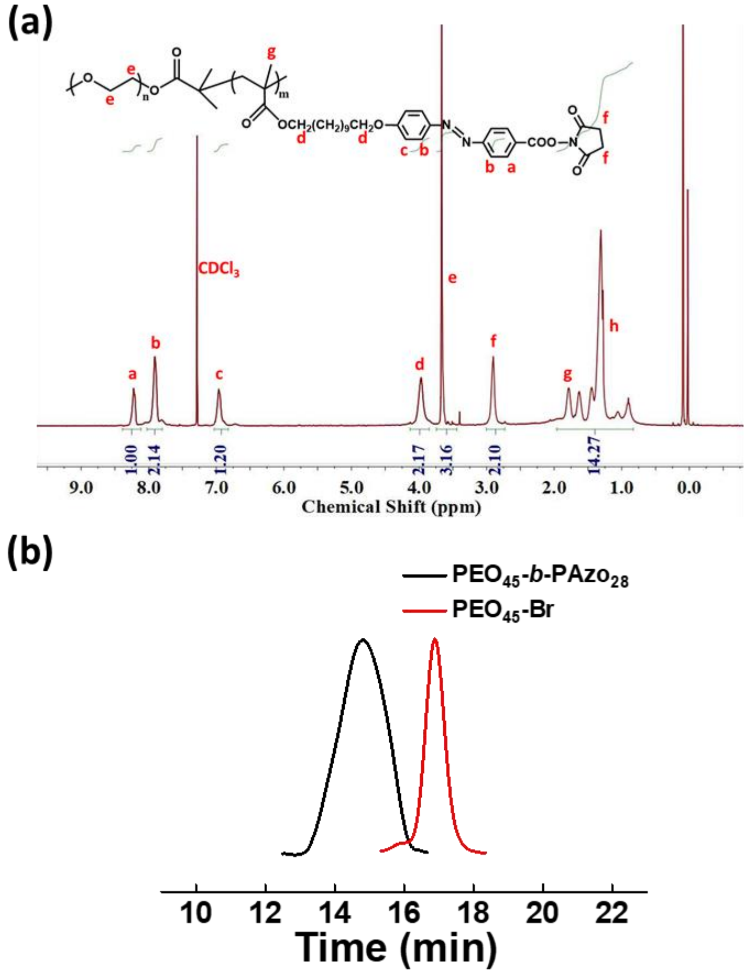

Figure 1. (a) The typical ^{1}H NMR spectrum of BCPs, using $CDCl_3$ as the solvent. (b) GPC traces of BCPs and macroinitiators (THF, polystyrene standards).

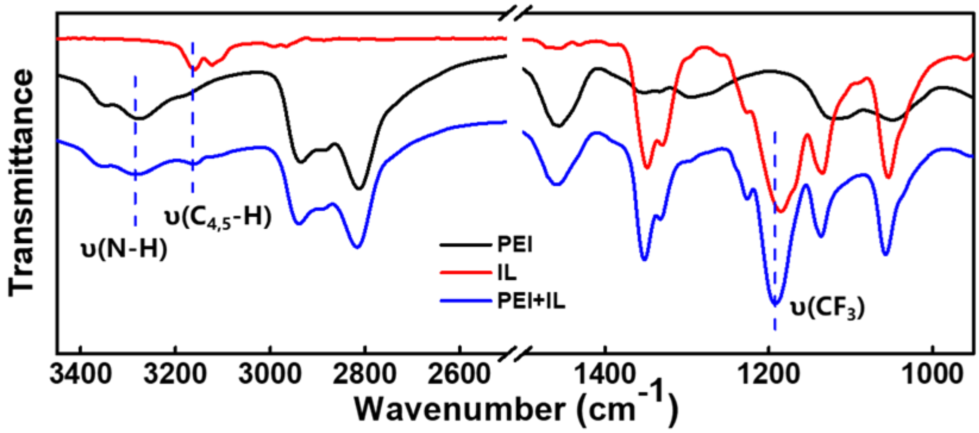

Figure 2. FTIR spectra of PEI, IL, and PEI/IL blends between 900 ~ 3500 cm^{-1}. The weight ratio of PEI/IL blends was 1:1. The shifting of N-H stretching bands from PEI, $C_{4,5}$-H stretching bands from cations, and C-F stretching bands from anions can prove the presence of electrostatic interactions and hydrogen bonds between ILs and PEI.

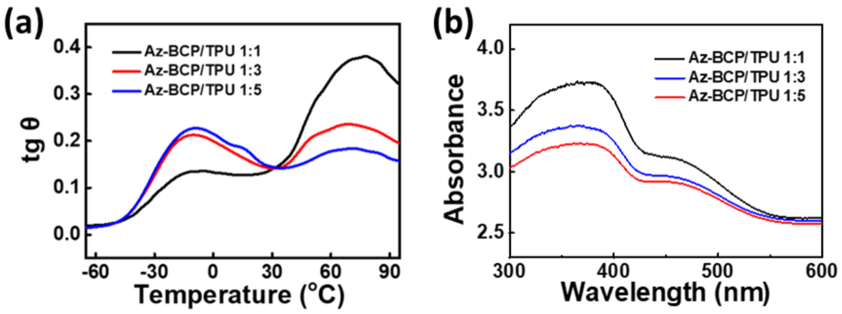

Figure 3. (a) Plots of tg θ *versus* temperature of Azo-BCP/TPU fabrics in different weight ratios. The two peaks of glass transition temperature (T_g) were fused in each weight ratios, which confirmed the good compatibility of Azo-BCP/TPU. (b) UV-Vis absorption spectra of Azo-BCP/TPU fabrics in different weight ratios. Film thickness: 5 μm.

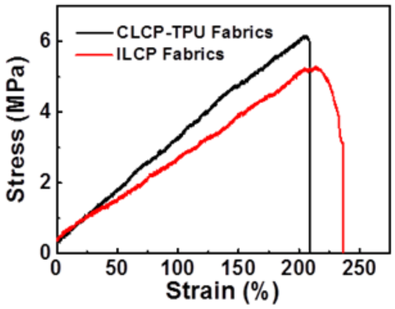

Figure 4. The stress-strain curves of the CLCP-TPU fabrics and ILCP fabrics.

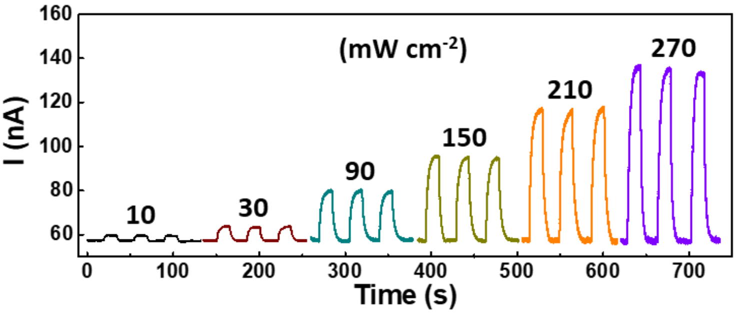

Figure 5. Initial data of current changes in accordance with UV power density from 10 to 270 mW cm^{-2}.

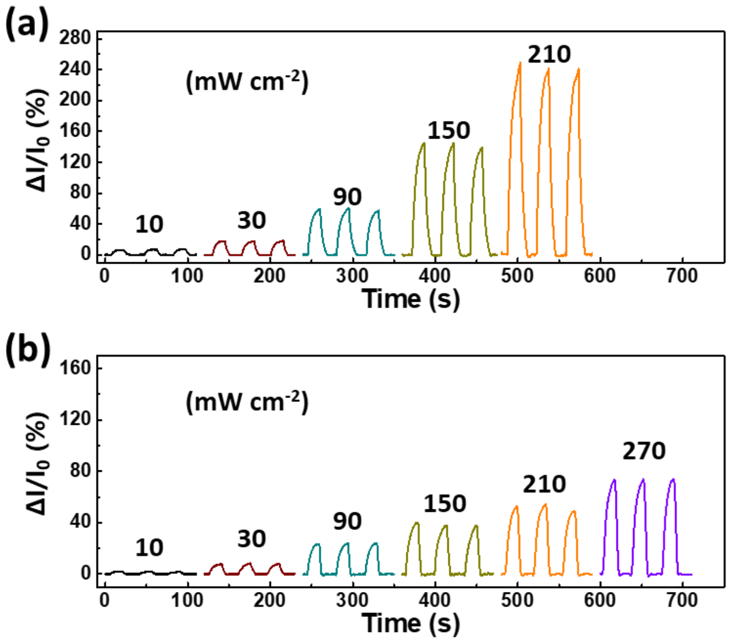

Figure 6. Relative current changes in accordance with UV power density from 10 to 270 mW cm^{-2}. The weight ratios of Azo-BCP/TPU in ILCPs are (a) 1:1 and (b) 1:5.

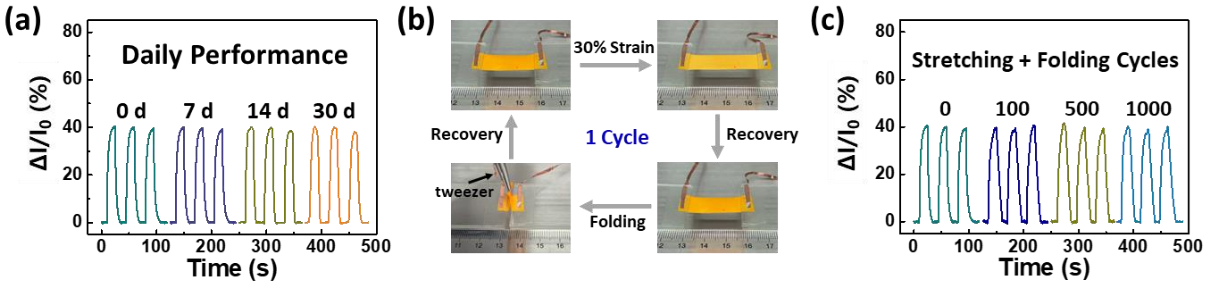

Figure 7. (a) The daily performance (0~30 days) of ILCP-based device at room temperature. (b) Schematic diagram of the consecutive stretching and folding cyclic operation. A 30% strain stretching operation plus a folding operation denotes as one cycle. The device was fixed on a linear motor to control the cyclic operation. A tweezer was used to fold the device. (c) The performance of ILCP-based device during consecutive 1000 stretching and folding cycles. The relative current changes were recorded upon 90 mW cm^{-2} UV light.

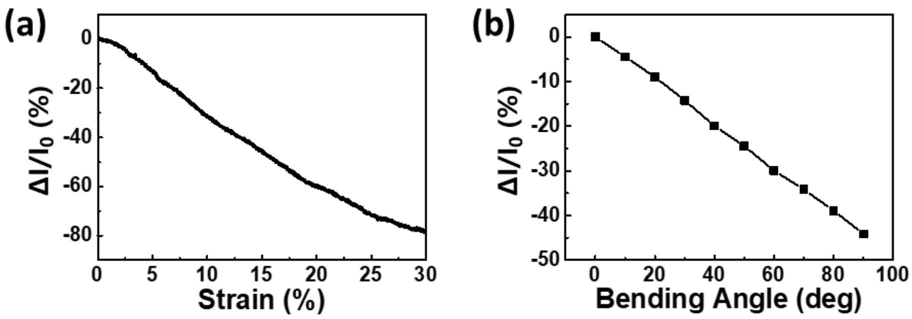

Figure 8. Relative current changes under (a) uniaxial 0~30% strain stretching and (b) 0~90° bending.

Table 1. The flexibility comparison of wearable photodetectors.

Active materials		Substrate	Flexibility	Response time	Ref.
Photoelectric materials	ZnO nanorod array	Ni textile	120° bending	6 s	[3]
	p-CuZnS/n-TiO_2	Ti microfiber	60° bending	< 0.2 s	[4]
	Reduced graphene oxide-ZnO	Mica	180° bending	17.9 s	[5]
	P3HT:PCBM	PET	180° bending	< 1 s	[6]
	Porphyrin-SWNT	PET	50% strain	~ 150 s	[7]
Functional polymers	Azobenzene/α-cyclodextrin	Hydrogel	90% strain	~ 20 s	[8]
	Azobenzene	PNIPAm IL gels	-	~ 6 min	[9]
	Diarylethene-containing ILs	PEO	-	~ 15 min	[10]
	Azo-CLCPs	Azo-CLCPs/TPU	240% strain/180° bending	5 s	This work

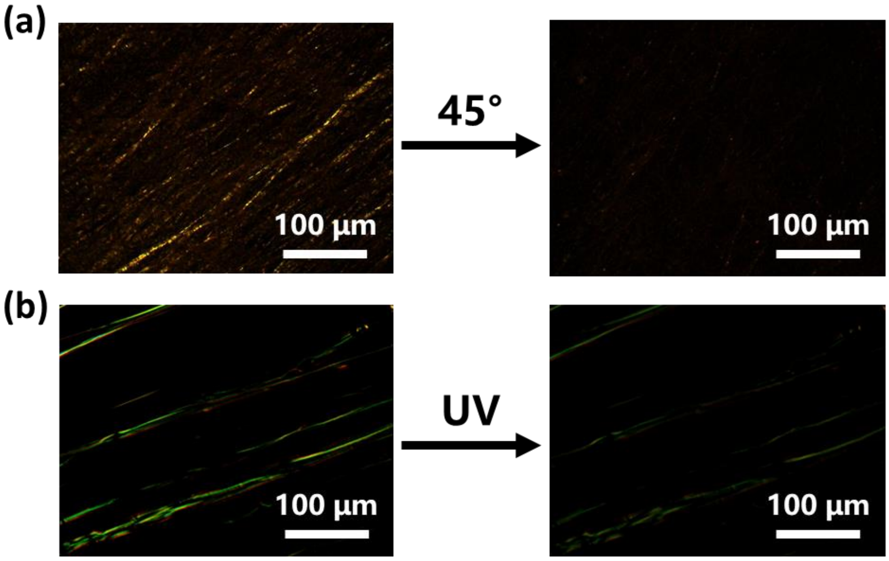

Figure 9. (a) POM images of the texture of the ILCP fibers before and after rotating the sample 45° with respect to the polarizer at room temperature. (b) POM images of the texture of the ILCP fibers before and after UV irradiation.

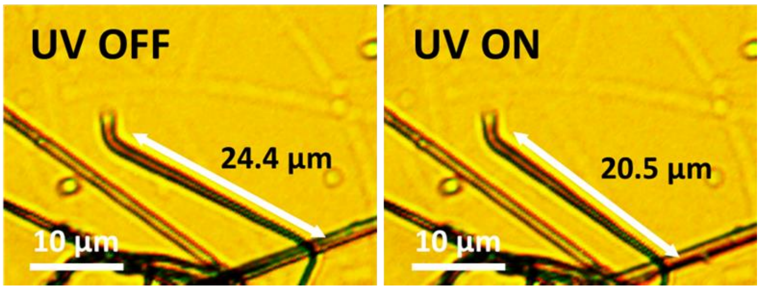

Figure 10. OM images of the single ILCP fiber before and after UV irradiation.

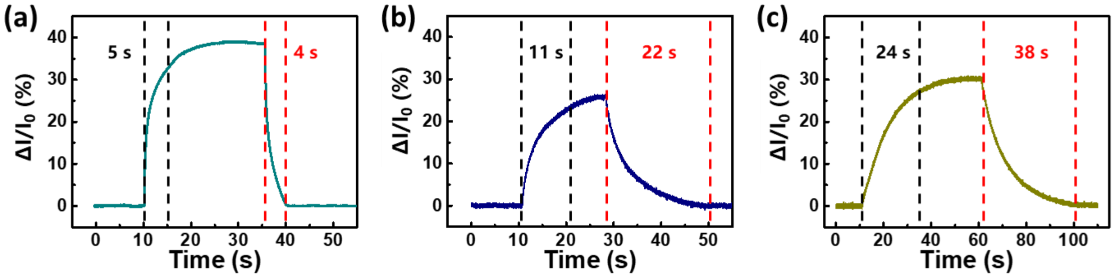

Figure 11. The response and recovery time of (a) CLCP-TPU fabrics, (b) CLCP-PAN fabrics, and (c) non-crosslinked LCP-TPU fabrics upon 90 mW cm^{-2} UV light.

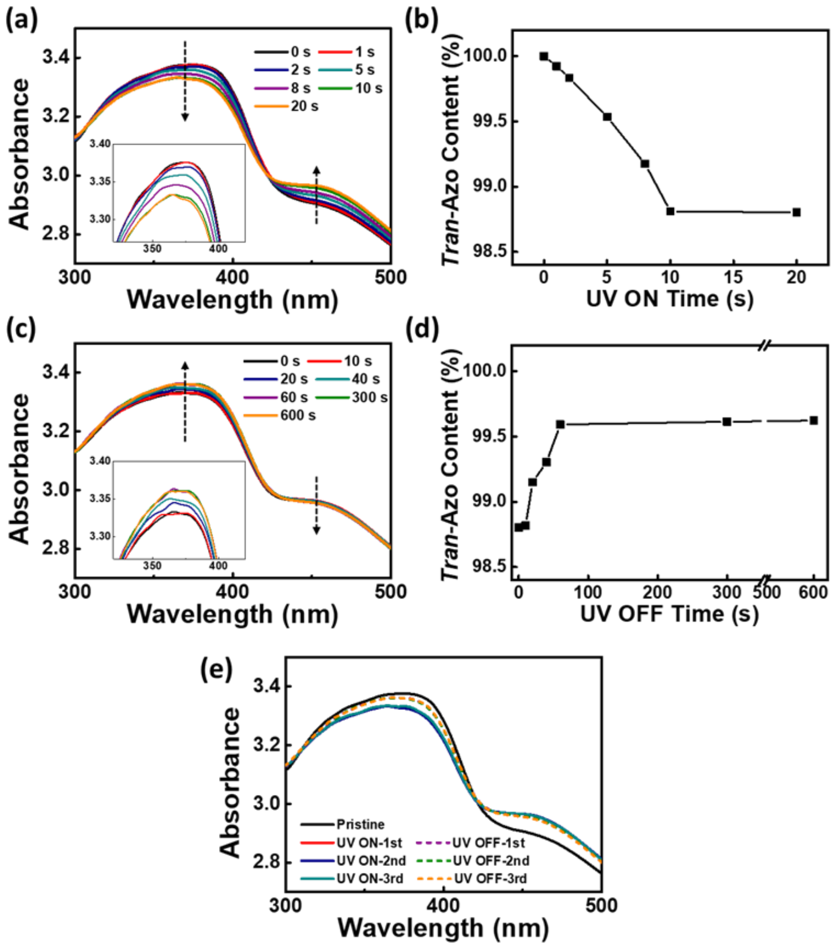

Figure 12. UV-Vis absorption spectra and plots of *trans*-Azo content *versus* time in the ILCP fabrics upon (a, b) turning on UV irradiation (10 mW/cm^{-2}) and (c, d) turning off UV irradiation, respectively. The *trans*-Azo content is defined as the ratio of absorption intensity over the pristine absorption intensity at 365 nm. (e) UV-Vis absorption spectra of 3 irradiation cycles. A cycle is consisted of 10 s of turning on UV irradiation and 60 s of turning off UV irradiation. Film thickness: 5 µm.

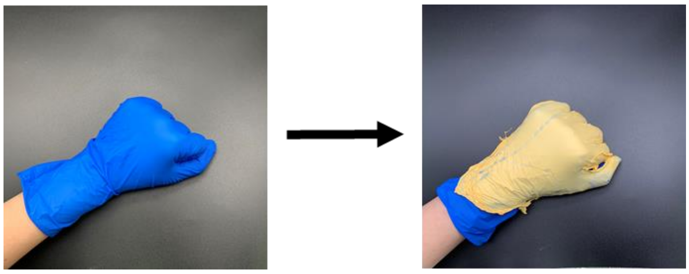

Figure 13. Photographs of the conformal and recyclable UV shielding LCP-TPU fabrics.

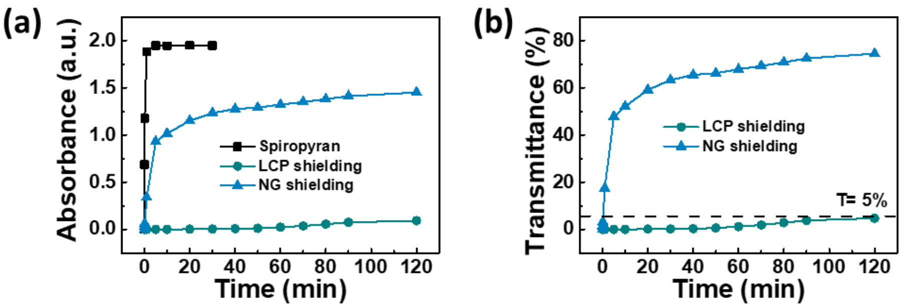

Figure 14. UV shielding property evaluation upon 20 mW cm^{-2} UV light by photochromic spiropyran indicator (10^{-5} mol/L, ethanol solution). (a) Plots of absorption spectra of spiropyran at 540 nm *versus* UV irradiation time. (b) Plots of the actual transmittance *versus* UV irradiation time. Define the actual transmittance as $T = A/A_{max}$, where A_{max} denote the max absorbance of spiropyran and A denote the absorbance of LCP shielding and NG shielding samples.

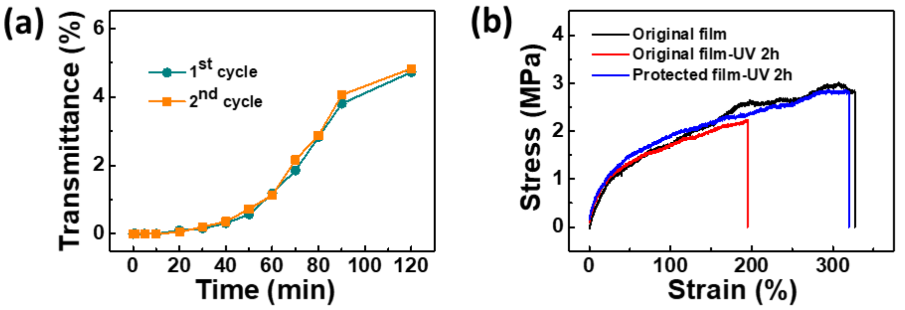

Figure 15. (a) Plots of the actual transmittance *versus* UV irradiation time of the 1st and 2nd cycles of LCP-TPU shielding samples. The LCP-TPU fabrics can be reused after irradiation with 520 nm visible light (200 mW cm^{-2}) for 10 min. (b) The stress-strain curves of the original nitrile film, the original nitrile film after 2 h UV irradiation, and the protected nitrile film after 2 h UV irradiation, respectively. The LCP-TPU fabrics can be applied to prevent the UV aging of nitrile gloves.

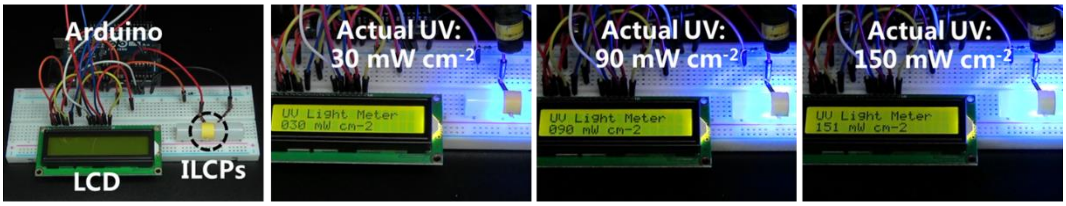

Figure 16. The physical connection diagram and practical working process of the LCD UV monitor.

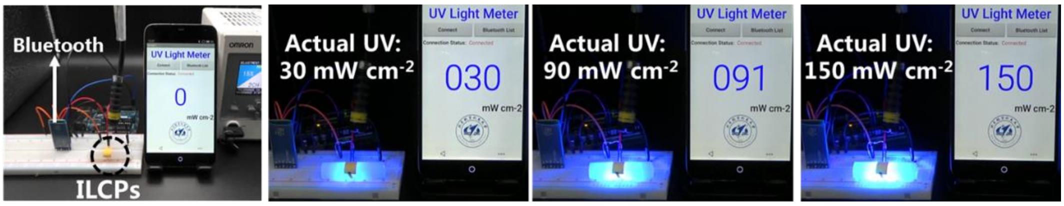

Figure 17. The physical connection diagram and practical working process of the remote Bluetooth UV monitor.

Section 3: Calculation of ultraviolet protection factor (UPF)

The calculation of UPF was based on the GB/T 18830-2009 evaluation method, which used the following equation:

$$\mathrm{UPF} = \frac{\sum_{\lambda=290}^{\lambda=400} E(\lambda) \times \varepsilon(\lambda) \times \Delta\lambda}{\sum_{\lambda=290}^{\lambda=400} E(\lambda) \times T(\lambda) \times \varepsilon(\lambda) \times \Delta\lambda}$$

Where λ is the wavelength, E(λ) is solar UV spectral irradiance, ε(λ) is the relative erythemal effectiveness, T(λ) is the spectral transmittance of the sample, and Δλ is the wavelength increment.

When UPF > 50 and UV-A light transmittance < 5%, the sample can be evaluated as UV protective textiles. To exclude the effect of film thickness, we defined the normalized UPF as the ratio of UPF over the film thickness.

Table 2. The calculated UPF of LCP-TPU fabrics (20 μm).

λ	E(λ)	ε(λ)	T(λ)		
			Original film	100% Strain	Washing
290	3.090E-06	1.000E+00	0.004	0.007	0.004
295	7.860E-04	1.000E+00	0.005	0.009	0.005
300	8.640E-03	6.490E-01	0.005	0.009	0.005
305	5.770E-02	2.200E-01	0.005	0.008	0.005
310	1.340E-01	7.450E-02	0.004	0.008	0.004
315	2.280E-01	2.520E-02	0.003	0.007	0.003
320	3.140E-01	8.550E-03	0.003	0.006	0.003
325	4.030E-01	2.900E-03	0.002	0.005	0.002
330	5.320E-01	1.360E-03	0.002	0.005	0.003
335	5.135E-01	1.150E-03	0.003	0.004	0.003
340	5.390E-01	9.660E-04	0.002	0.004	0.002
345	5.345E-01	8.100E-04	0.002	0.004	0.002
350	5.590E-01	6.840E-04	0.002	0.004	0.002
355	6.080E-01	5.750E-04	0.002	0.004	0.002
360	5.640E-01	4.840E-04	0.002	0.004	0.002
365	6.830E-01	4.070E-04	0.002	0.004	0.002
370	7.660E-01	3.430E-04	0.002	0.004	0.002
375	6.635E-01	2.880E-04	0.002	0.004	0.003
380	7.540E-01	2.430E-04	0.002	0.004	0.003
385	6.055E-01	2.040E-04	0.002	0.004	0.002
390	7.570E-01	1.720E-04	0.003	0.004	0.003
395	6.680E-01	1.450E-04	0.002	0.005	0.003
400	1.010E+00	1.220E-04	0.002	0.005	0.002
	UPF		25040	13851	24869
	Normalized UPF		1250	693	1243

Table 3. The calculated UPF of commercial UV protection clothing (90 μm).

λ	E(λ)	ε(λ)	T(λ)		
			Original film	100% Strain	Washing
290	3.090E-06	1.000E+00	0.059	0.289	0.141
295	7.860E-04	1.000E+00	0.068	0.299	0.145
300	8.640E-03	6.490E-01	0.079	0.306	0.148
305	5.770E-02	2.200E-01	0.087	0.313	0.152
310	1.340E-01	7.450E-02	0.093	0.327	0.158
315	2.280E-01	2.520E-02	0.100	0.337	0.162
320	3.140E-01	8.550E-03	0.104	0.347	0.168
325	4.030E-01	2.900E-03	0.111	0.351	0.170
330	5.320E-01	1.360E-03	0.118	0.355	0.172
335	5.135E-01	1.150E-03	0.124	0.358	0.173
340	5.390E-01	9.660E-04	0.129	0.360	0.174
345	5.345E-01	8.100E-04	0.134	0.362	0.176
350	5.590E-01	6.840E-04	0.137	0.365	0.177
355	6.080E-01	5.750E-04	0.141	0.366	0.177
360	5.640E-01	4.840E-04	0.143	0.369	0.179
365	6.830E-01	4.070E-04	0.145	0.375	0.181
370	7.660E-01	3.430E-04	0.148	0.377	0.183
375	6.635E-01	2.880E-04	0.154	0.385	0.186
380	7.540E-01	2.430E-04	0.157	0.387	0.187
385	6.055E-01	2.040E-04	0.159	0.388	0.190
390	7.570E-01	1.720E-04	0.161	0.390	0.193
395	6.680E-01	1.450E-04	0.164	0.392	0.192
400	1.010E+00	1.220E-04	0.168	0.395	0.193
UPF			1044	306	631
Normalized UPF			12	3.4	7.0

Table 4. The calculated UPF of nitrile gloves (80 μm).

λ	E(λ)	ε(λ)	T(λ)		
			Original film	100% Strain	Washing
290	3.090E-06	1.000E+00	0	0	0
295	7.860E-04	1.000E+00	0.001	0.001	0.001
300	8.640E-03	6.490E-01	0.002	0.003	0.002
305	5.770E-02	2.200E-01	0.002	0.004	0.002
310	1.340E-01	7.450E-02	0.002	0.003	0.002
315	2.280E-01	2.520E-02	0.002	0.004	0.002
320	3.140E-01	8.550E-03	0.003	0.004	0.003
325	4.030E-01	2.900E-03	0.003	0.006	0.003
330	5.320E-01	1.360E-03	0.003	0.007	0.003
335	5.135E-01	1.150E-03	0.005	0.009	0.006
340	5.390E-01	9.660E-04	0.006	0.012	0.007
345	5.345E-01	8.100E-04	0.008	0.015	0.009
350	5.590E-01	6.840E-04	0.009	0.019	0.010
355	6.080E-01	5.750E-04	0.011	0.023	0.013
360	5.640E-01	4.840E-04	0.015	0.029	0.017
365	6.830E-01	4.070E-04	0.019	0.038	0.022
370	7.660E-01	3.430E-04	0.026	0.052	0.030
375	6.635E-01	2.880E-04	0.038	0.074	0.044
380	7.540E-01	2.430E-04	0.052	0.099	0.060
385	6.055E-01	2.040E-04	0.067	0.125	0.078
390	7.570E-01	1.720E-04	0.086	0.155	0.100
395	6.680E-01	1.450E-04	0.109	0.189	0.127
400	1.010E+00	1.220E-04	0.132	0.222	0.153
UPF			24250	13407	22351
Normalized UPF			303	168	279

References

[1] Tian, Y. *et al.* Synthesis, Nanostructures, and Functionality of Amphiphilic Liquid Crystalline Block Copolymers with Azobenzene Moieties. *Macromolecules* **35**, 3739 (2002).

[2] Lv, J. *et al.* A reactive azobenzene liquid-crystalline block copolymer as a promising material for practical application of light-driven soft actuators. *J. Mater. Chem. C* **3**, 6621 (2015).

[3] Zhu, Z. *et al.* Improving Wearable Photodetector Textiles via Precise Energy Level Alignment and Plasmonic Effect. *Adv. Electron. Mater.* **3**, 1700281 (2017).

[4] Xu, X. *et al.* A Real-Time Wearable UV-Radiation Monitor based on a High-Performance p-CuZnS/n-TiO_2 Photodetector. *Adv. Mater.* **30**, 1803165 (2018).

[5] An, J. *et al.* Single-step selective laser writing of flexible photodetectors for wearable optoelectronics. *Adv. Sci.* **5**, 1800496 (2018).

[6] Park, S. H. *et al.* 3D printed polymer photodetectors. *Adv. Mater.* **30**, 1803980 (2018).

[7] Pyo, S. *et al.* Heterogeneous Integration of Carbon-Nanotube-Graphene for High-Performance, Flexible, and Transparent Photodetectors. *Small* **13**, 1700918 (2017).

[8] Wang, H. *et al.* Reversible Ion-Conducting Switch in a Novel Single-Ion Supramolecular Hydrogel Enabled by Photoresponsive Host-Guest Molecular Recognition. *Adv. Mater.* **31**, 1807328 (2019).

[9] Wang, C. *et al.* Reversible Ion-Conducting Switch by Azobenzene Molecule with Light-Controlled Sol-Gel Transitions of the PNIPAm Ion Gel. *ACS Appl. Mater. Interfaces* **12**, 42202 (2020).

[10] Nie, H. *et al.* Light-Controllable Ionic Conductivity in a Polymeric Ionic Liquid. *Angew. Chem. Int. Ed.* **59**, 5123 (2020).
