# Supplementary material for: Azobenzene-containing liquid crystalline composites for robust ultraviolet detectors based on conversion of illuminance-mechanical stress-electric signals
Source: Nat Commun. 2021 Aug 12;12:4875. doi: 10.1038/s41467-021-25178-2 (PMC8360969; doi:10.1038/s41467-021-25178-2)
Supplement: Supplementary file 3 — Description of Additional Supplementary Files [file 41467_2021_25178_MOESM3_ESM.pdf]

Description of Additional Supplementary Files

File Name: Supplementary Movie 1
Description: Adhesion ability of ILCP fabrics to skins.

File Name: Supplementary Movie 2
Description: OM and POM images of the deformation of ILCP fibers upon UV light.

File Name: Supplementary Movie 3
Description: The practical working process of the UV monitors through LCD screen and remote Bluetooth.

File Name: Supplementary Movie 4
Description: The practical working process of UV security unlock

File Name: Supplementary Movie 5
Description: The practical working process of UV security input.
